# Optical coherence tomography: when a picture is worth a million words

**DOI:** 10.1172/JCI174951

**Published:** 2023-09-21

**Authors:** Simone Tzaridis, Martin Friedlander

**Affiliations:** 1 Department of Molecular Medicine, The Scripps Research Institute, La Jolla, California, USA;; 2 The Lowy Medical Research Institute, La Jolla, California, USA.; 3 Department of Ophthalmology, University Hospital of Bonn, Bonn, Germany.; 4 Division of Ophthalmology, Scripps Clinic, La Jolla, California, USA.

The 2023 Lasker-DeBakey Clinical Medical Research Award has been awarded to James G. Fujimoto (Massachusetts Institute of Technology [MIT]), David Huang (Oregon Health and Science University–Casey Eye Institute), and Eric A. Swanson (MIT) for the invention of optical coherence tomography (OCT). While this technology has substantially enhanced our ability to image a variety of tissues in multiple organ systems, perhaps its greatest impact to date has been in the field of ophthalmology. The eye is truly a window into the rest of the human body and, as such, provides the astute clinician an intimate view of the vasculature, neuronal tissue, glia, and even immunocytes. Properly viewed and analyzed, images of the anterior (e.g., cornea, iris, lens, and conjunctiva) and posterior (e.g., vitreo-retinal interface, retina, choroid, and optic nerve head) segments of the eye can be used to diagnose, assess response to therapy, and record the progression of ocular as well as systemic diseases. While more established ocular imaging modalities (e.g., fundus photography, fluorescein angiography, magnetic resonance imaging, ultrasonography) provide very useful information, high-resolution, longitudinal *z*-series imaging of live tissue had remained elusive until the invention and application of OCT.

OCT has most profoundly affected the ophthalmic subspecialties of retina and glaucoma. The ability to optically dissect a living retina and optic nerve head in real time at up to 3 μm resolution has revolutionized the manner in which we image the eye (Figure 1). Such images cannot be obtained in living tissue with any other imaging modality. In ophthalmology, OCT has become a standard of care, impacting the lives of millions of patients every year. The interest in and importance of OCT in clinical research and development is also reflected by increasing numbers of scientific publications. First described in the literature in 1991 in a *Science* paper by Huang et al. (1), OCT has since been the subject of more than 64,000 scientific publications. Approximately 30 million OCT imaging procedures are performed every year on 50,000 OCT systems worldwide (2, 3).

## OCT for dummies: transforming light into quasi-histological images

Originating from the field of femtosecond optics (4), OCT uses low-coherence interferometry to generate cross-sectional images of tissue structures with a resolution on the (sub-)micron scale and an image penetration depth of several millimeters (1). The main mode of action is comparable to that of ultrasound, however, OCT uses a reflection of light instead of sound waves. Optically accessible biological structures can be scanned using a beam of light, and then the backscatter or reflectance is recorded. More precisely, this technique records and compares the optical path length of received photons to a known reference path of traveling light, while photons that scatter multiple times before detection are rejected as “background noise.” Thus, OCT can generate reflectivity profiles (“A-scans”) by subtracting any background scatter and recording light that is directly reflected from ocular or other structures of interest (1, 5, 6). These axial scans contain information about the location and spatial dimensions of a structure. By moving the light beam laterally across the structure of interest, a cross-sectional tomogram (B-scan) can be generated. Several B-scans can then be combined into a three-dimensional image (1, 5, 6).

## Fujimoto, Huang, and Swanson shape the early history of OCT

Several research groups initiated studies in the 1980s on low-coherence, partial coherence, or white-light interferometry for in vivo imaging, particularly of ocular structures (4, 7, 8). A major breakthrough came in 1991 when David Huang, working in Fujimoto’s laboratory at MIT, demonstrated the feasibility of using low-coherence interferometry for in vitro imaging of the peripapillary area of the retina and coronary artery, coining the term “optical coherence tomography” (1). In 1993, Swanson et al. published the first in vivo OCT images of the retina (9).

The invention and translation of OCT from basic research and early in vitro studies to a standard-of-care device required, however, multidisciplinary expertise and collaborations across multiple fields. Commercialization and entrepreneurship were critical factors in OCT’s impact on clinical care. The key technologies that Fujimoto and his team developed and patented eventually led to OCT devices for clinical use (2, 3). Over the years, the technology has evolved and been refined, improving image resolution, acquisition speed, and penetration depth and has led to novel applications.

These improvements were enabled by the development of spectral-domain and swept-source OCT, both subsets of Fourier-domain detection. Early OCT devices used time-domain detection, an interferometer with a scanning reference arm, and low-coherence light. With the advent of spectral-domain OCT, a system using a stationary reference arm, sensitivities could be substantially increased, measuring all scatters of light simultaneously (3, 6). With further advances in laser technology development, swept-source OCT enabled a significant increase in imaging speed without the need of a spectrometer or line scan cameras (3). Furthermore, operating at long wavelengths, swept-source OCT provides enhanced-depth imaging, enabling, for instance, detailed imaging of the choroid (the most posterior vascular plexus in the eye) (6, 10). Further examples of recent developments include Doppler-OCT and OCT-angiography, respectively, which provide detailed, noninvasive flow imaging of blood vessels without the need for intravascular dyes. In 2007, Huang and his group demonstrated the first in vivo measurements of total retinal blood flow using Fourier-domain Doppler-OCT (11).

## OCT in ophthalmic imaging

Systemic disease is not infrequently diagnosed during a dilated eye exam; direct visualization of the retina and optic nerve head provide a glimpse of the brain, while the state of the systemic vasculature (e.g., hypertension, atherosclerotic plaques) may be assessed by viewing retinal blood vessels. The most common intraocular tumor in women is metastatic breast carcinoma, and retinal metastases may be the first indication of the disease if central vision is affected. Direct and indirect ophthalmoscopy provide the means to image neuronal, vascular, and glial structures in the retina. These images may be recorded on static photographs, but these only capture the surface of the structures being imaged. OCT changed that dramatically by making it possible to capture a “*z*-series” through the retina and optic nerve head as well as to acquire enormous amounts of digital data that can be computationally analyzed, making it possible for the first time to track and quantify pathological changes in a meaningful way.

## The impact of OCT on assessing and managing ophthalmic disease

**Figure 1:**
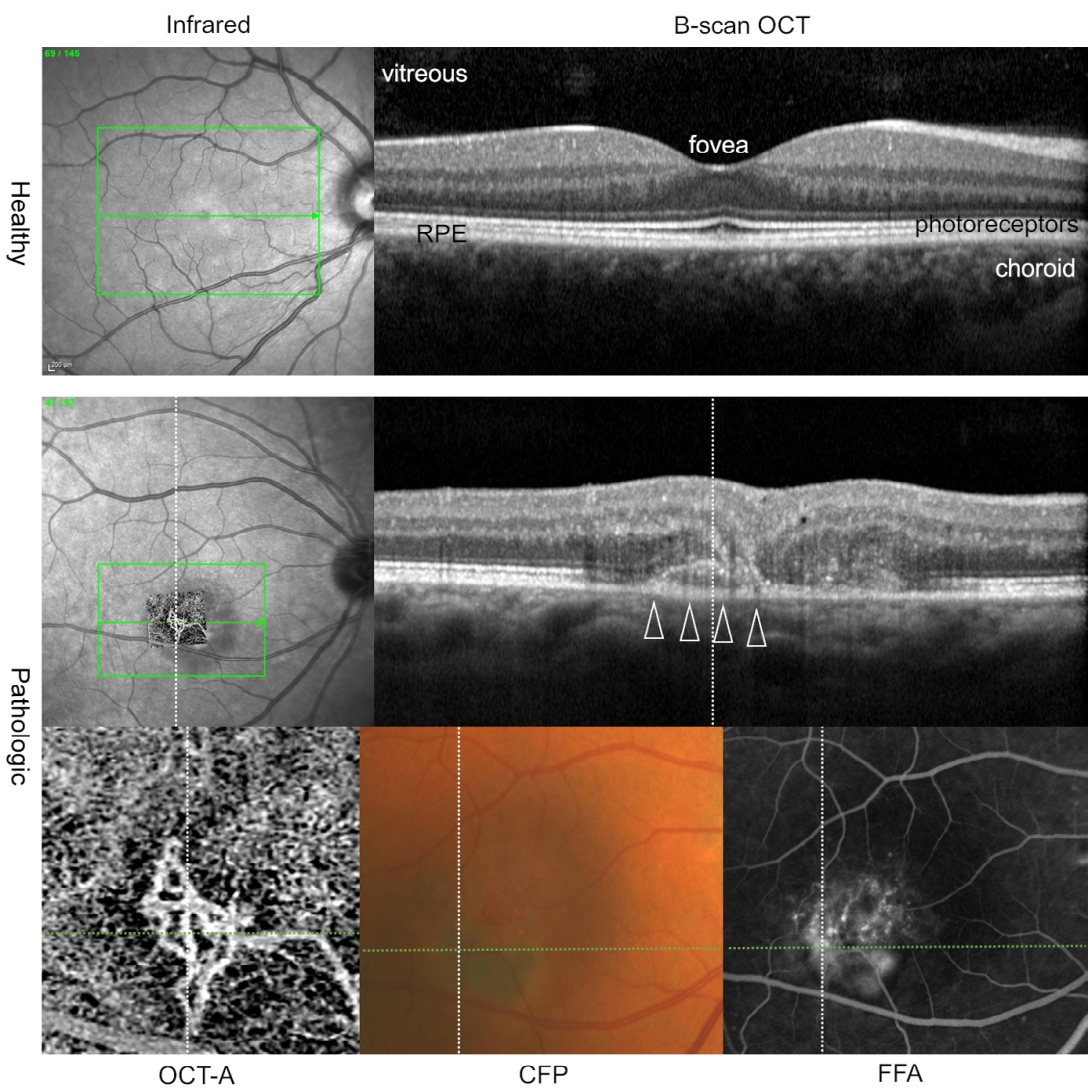
OCT provides highly detailed images of the central retina in a healthy eye and a diseased eye. OCT B-scans permit the differentiation of retinal layers, the retinal pigment epithelium (RPE), and choroid in the central retina in healthy and diseased eyes. Horizontal lines on en face images (infrared images; OCT-angiography [OCT-A] of the outer retina-choriocapillaris layer; color fundus photography [CFP]; and fundus fluorescein angiography [FFA]) indicate the exact position of each OCT B-scan. Note the additional information OCT images provide, in comparison with conventional imaging methods (CFP and FFA, bottom row), including the clear delineation of a neovascular membrane (white arrowheads). OCT-A facilitates imaging of retinal and choroidal blood flow, enabling a detailed illustration of the neovascularization (bottom row: enlarged OCT-A image of neovascularization; middle row: projection of the OCT-A en face image on the infrared image). Written informed consent was received from participants to use their deidentified images.

The vast majority of diseases that cause catastrophic loss of vision do so as a result of vascular changes leading to hemorrhage, edema, and significant disruption of the normal retinal architecture. Similarly, neurodegeneration can lead to loss of visual function in association with vascular and glial abnormalities. Although earlier direct imaging modalities enabled evaluation of the retinal surface, OCT provided a window into the depths of the retina and optic nerve head. OCT offered a whole new way to quantitatively evaluate the extent of edema and structural alterations. With an axial resolution on the micron scale, it became possible to determine which of the nine retinal layers were affected by a disease, thus enabling a better diagnosis and prognosis. Figure 1 shows an example of a healthy eye and a diseased eye imaged with OCT, OCT-angiography, and conventional en face imaging, respectively. With the development of VEGF antagonists and their introduction into the clinics for the treatment of neovascular eye diseases like “wet” macular degeneration and diabetic retinopathy, a rapid imaging technique was needed to quantify retinal thickness resulting from neovascular edema. OCT made this possible and became critical in managing patients on VEGF antagonists, and it continues to serve as the chief method to monitor these patients.

OCT has also greatly impacted the field of glaucoma, a progressive, blinding disease of the eye that is due to the loss of ganglion cells of the inner retina. Glaucoma commonly begins with peripheral vision loss, gradually progressing to loss of central vision in its end stages. Progression of the disease and its response to therapy can now be readily quantified, rapidly assessed, and managed using OCT in a manner that was not available prior to OCT.

In addition to recording and quantifying retinal changes in three dimensions, OCT imaging can also be used as a surrogate marker for visual function. For example, in macular telangiectasia, disruption of the inner segment/outer segment layer (or “IS/OS break”) correlates in a highly statistically meaningful fashion with loss of photoreceptor function as determined by microperimetry (central visual field mapping at high resolution) (12, 13). In diseases in which visual acuity is not an accurate assessment of visual function, this becomes an important way to rapidly assess the progression of disease or response to therapy (13–16). In trials designed to assess neuroprotection in neurodegenerative diseases such as macular telangiectasia, measuring the extent of the IS/OS break using OCT is a useful clinical endpoint for loss of photoreceptors (16).

OCT has also proven to be highly valuable as a research tool. Defining entry criteria and outcome measures for clinical trials can determine the efficacy of a treatment or investigational product. OCT has been used to determine exclusion criteria that identify patients who will be less likely to benefit from a specific therapeutic intervention and as a way to quantify clinical endpoints in a trial measuring a drug’s effect on the progression of neurodegeneration (15). While OCT is still mainly used in clinical settings, prototype OCT devices are also being developed for at-home monitoring of patients (17).

OCT imaging is particularly well suited for the future application of artificial intelligence and deep-learning algorithms (18). Examples include the automated quantitative analysis of OCT images that enables a detailed segmentation of retinal layers or analysis of specific disease features such as changes in retinal thickness or the presence of intraretinal fluid (19). Further applications include the automated identification and prediction of disease progression and treatment response for retinal diseases, and other novel applications are rapidly emerging (20, 21).

## Applications beyond ophthalmology

Outside of the eye, OCT has a number of applications, providing an in vivo “virtual biopsy” of various tissues and organs. Luminal organs, such as vessels, airways, or the esophagus, as well as larger hollow organs, such as the stomach, cervix, or urinary bladder, can be evaluated using OCT (22). In the field of cardiology, OCT can be used to guide coronary interventions, providing clear luminal images of coronary vessels. Types of atherosclerotic plaques can be differentiated and the vascular response after coronary stent implantation can be monitored using OCT (23). Endobronchial OCT can be used to assess the structures of airway walls, quantify airway lumen caliber and compliance, and detect diseased areas in patients with lung cancer, for example (24). In gastroenterology, applications of OCT include the detection of dysplasia of the esophagus, imaging of colon polyps, and screening for colon cancer. In dermatology, OCT can be used to evaluate skin morphology, assist with diagnosing skin diseases including nonmelanoma skin cancer, monitor inflammatory diseases, and evaluate the response to treatment. Lymph nodes and pathological changes in lymph node morphology due to malignancies can be visualized using OCT, thus providing oncologic applications (25). Early signs of neurological and neurodegenerative diseases, such as Alzheimer’s, Parkinson’s, or multiple sclerosis, may be evaluated using retinal/peripapillary OCT scans.

## Closing remarks

OCT has revolutionized the diagnosis and management of patients with ophthalmic disease. It has facilitated studies of the eye that could not have otherwise been done. It has enabled us to “stage” ophthalmic disease on the basis of real-time imaging of ocular structures that, prior to OCT, were only visualized postmortem in fixed, sectioned tissue. This has not only proven highly valuable when designing clinical trials as discussed above, but also in monitoring the response to therapy. This ability to detect the most subtle pathologic changes, such as the smallest amounts of intra- and subretinal fluid, is both a blessing and a curse. A blessing because it allows us to diagnose a disease and determine disease progression at earlier stages than ever before, and a curse because not all of the changes we detect with OCT are functionally relevant and require treatment. In order to avoid overtreatment and an unnecessary increase in treatment burden, OCT images need to be cautiously interpreted in conjunction with the overall clinical picture, other imaging modalities, and measures of function. OCT-based machine-learning algorithms may, in the near future, be able to assist with tailoring individual treatment regimens to patients and bring us closer to individualized precision medicine (26, 27).

A picture may be worth a thousand words, but an OCT image is worth a million! As the engineers continue to endow OCT with higher resolution and increased penetrability, the extensive use of this technology has brought the cost down to the point where it is readily accessible to most patients. Serial imaging with this remarkable instrument has vastly improved the clinician’s ability to study and examine the eye and, in so doing, blunted the progression of a multitude of ocular conditions that previously led to loss of vision.

## Version 1 09/21/2023

Electronic publication

## Version 2 09/29/2023

Electronic publication
